# Dietary Intake Is Similar Among Adult Men with Different Levels of Cold-Induced Brown Adipose Tissue Activation

**DOI:** 10.3390/nu16213697

**Published:** 2024-10-30

**Authors:** Andres E. Carrillo, Petros C. Dinas, Argyro Krase, Eleni Nintou, Alexandros Georgakopoulos, Marinos Metaxas, Edward J. Ryan, Maria Vliora, Panagiotis Georgoulias, Sofia Chatziioannou, Andreas D. Flouris

**Affiliations:** 1 Department of Exercise Science, College of Health Sciences, Chatham University, Pittsburgh, PA 15232, USA; acarrillo@chatham.edu (A.E.C.); eryan@chatham.edu (E.J.R.); 2 FAME Laboratory, Department of Physical Education and Sport Science, University of Thessaly, 42100 Trikala, Greece; petros.cd@gmail.com (P.C.D.); argyrokrase@hotmail.com (A.K.); enintou@gmail.com (E.N.); mvliora@gmail.com (M.V.); 3 PET/CT Department, Biomedical Research Foundation, Academy of Athens, 11527 Athens, Greece; ageorgakopoulos727@gmail.com (A.G.); mmetaxas@bioacademy.gr (M.M.); sofiac@med.uoa.gr (S.C.); 4 Nuclear Medicine Laboratory, Faculty of Medicine, University of Thessaly, 41334 Larisa, Greece; pgeorgoul@med.uth.gr

**Keywords:** brown fat, BAT, thermogenesis, energy expenditure, energy balance, body composition, nutrition, macronutrients

## Abstract

Background/Objectives: Brown adipose tissue (BAT) activation has important metabolic health implications, yet the relationship between habitual dietary intake and BAT activity in humans remains to be fully understood. Methods: We compared dietary intake among adult men with (BAT_positive_, age: 34.8 ± 5.4 years, BMI: 28.2 ± 5.3 kg/m^2^, *n* = 12) and without (BAT_negative_, age: 39.1 ± 4.1 years, BMI: 31.1 ± 6.7 kg/m^2^, *n* = 11) cold-induced BAT activation. Activation of BAT was measured immediately following 2 h of cold exposure using ^18^F fluorodeoxyglucose positron emission tomography and computed tomography reported as maximum standardized uptake (SUV_max_). Participants categorized as BAT_positive_ had an SUV_max_ > 1.5 g/mL that was normalized to lean body mass (SUV_lean_) for analysis. Shivering intensity was recorded every 15 min during cold exposure and dietary intake was estimated from 7 consecutive 24 h dietary recalls. Results: The BAT_negative_ group was significantly older than the BAT_positive_ group (*p* = 0.046). Although BAT_negative_ participants consumed an average of 281.2 kcal/day more than BAT_positive_, there were no significant differences in dietary intake between groups (*p* ≥ 0.202). Further, no statistically significant associations between SUV_lean_ and dietary intake among BAT_positive_ participants were observed (*p* ≥ 0.175). Participants who shivered (*n* = 9) during cold exposure tended to be shorter (*p* = 0.056) and have a lower waist-to-hip ratio (*p* = 0.097) but did not differ in dietary intake (*p* ≥ 0.204) or BAT activity (*p* = 0.964) when compared to the non-shivering (*n* = 11) group. Conclusions: Our results indicate that BAT activity and shivering during cold exposure are more strongly related to variables such as age and body size or composition rather than habitual dietary intake. We conclude that habitual dietary intake likely has a negligible influence on BAT activity among adult men.

## 1. Introduction

Activation of brown adipose tissue (BAT) results in heat production that contributes to body temperature regulation during cold exposure [1]. Heat production via BAT activation, referred to as non-shivering thermogenesis, is stimulated when the sympathetic nervous system initiates interaction between norepinephrine and adrenergic receptors on brown adipocytes [2]. Non-shivering thermogenesis occurs when uncoupling protein 1 (UCP1), a BAT-specific protein embedded within the inner mitochondrial membrane, facilitates dissipation of energy in the form of heat—a process uncoupled from the synthesis of adenosine triphosphate [3]. Brown adipose tissue is conveniently located in the core region (e.g., neck) of the body, providing ample heat to critical areas when stimulated during cold exposure [4,5]. The considerable metabolic activity of UCP1 in BAT mitochondria has resulted in the exploration of clinical implications, such as body weight and blood glucose regulation [6,7,8,9]. For example, higher chronological age, overweight or obesity, and the presence of chronic disease have been associated with lower levels of BAT mass and/or activity [1,10,11]. More recently, in an attempt to further understand the potential malleability of BAT, others have investigated characteristics of BAT function in relation to lifestyle factors, such as physical activity and diet [6,10,12,13,14].

The relationship between dietary intake and BAT has been explored in several contexts [15,16,17]. For example, BAT-dependent diet-induced thermogenesis (DIT) and postprandial metabolic activity of BAT have been studied to further understand the potential impacts on body weight regulation and metabolic health. To investigate whether a carbohydrate-rich, high calorie meal activates BAT, Vosselman et al. [12] assessed postprandial BAT activity via ^18^F fluorodeoxyglucose (^18^F-FDG) positron emission tomography-computed tomography (PET/CT) in 11 young men and reported increased postprandial glucose uptake in BAT but found no relationship between BAT activity and DIT. Findings from a meta-analysis conducted by our group, revealed no mean differences in standardized uptake value (SUV) of BAT following a standard meal, a carbohydrate-rich, high calorie meal, or after overfeeding [13]. Other relevant investigations have explored the effects of consuming particular foods or food items, such as capsinoids, on BAT activity [18,19,20,21]. Specifically, in a cross-over design, 20 young adult men and women underwent an ^18^F-FDG PET scan to measure BAT volume and activity after 12 g of capsinoid ingestion or cold exposure. Participants were categorized as either BAT-positive (SUV ≥ 2 g/mL) or BAT-negative (SUV < 2 g/mL) depending on cold-induced BAT activation levels. Capsinoid ingestion did not result in detectable BAT activation but did lead to greater increases in energy expenditure among BAT-positive compared to BAT-negative participants [19].

More recently, longer-term influences of a particular diet or habitual macronutrient intake have been investigated in relation to BAT mass and activation [22,23,24]. For example, a ketogenic diet fed to mice for 4 weeks resulted in larger BAT mitochondrial size and increased UCP1 levels when compared to mice fed standard chow [25]. Others have shown that omega-6 and -3 polyunsaturated fatty acid intake in mice are associated with increased thermogenic activity of BAT [26,27,28]. Studies in humans, however, have shown some inconsistency [22]. Our group, and others, reported that general dietary habits appear to have a negligible influence on BAT activity and browning formation markers in subcutaneous white adipose tissue [13,23,29]. In 102 young adults, Sanchez-Delgado et al. [23] found no association between cold-induced BAT ^18^F-FDG uptake and habitual energy intake estimated from three non-consecutive 24-h dietary recalls. One of the first studies to investigate associations between dietary factors and BAT activity in humans showed that energy density, ethanol intake, and specific dietary patterns (e.g., Mediterranean diet, pro-inflammatory diet) were weakly associated with cold-induced BAT activity [24]. Nevertheless, the relationship between chronic dietary factors or dietary habits and BAT activity in humans remains to be fully understood.

The aim of this study was to compare dietary intake among adult males with different levels of cold-induced BAT activation. A secondary aim was to assess the relationship between dietary factors, magnitude of BAT activation, and shivering in response to cold exposure.

## 2. Materials and Methods

This study is part of a larger clinical trial (clinical trial registration number: NCT0403737) that examined the association between the thermogenic activity of subcutaneous white adipose tissue and environmental temperature [30]. This study conformed to the standards set by the Declaration of Helsinki and was approved by the ethics committees at the University of Thessaly (protocol number: 1297/6-12-2017) and the Biomedical Research Foundation at the Academy of Athens, (protocol number: 7/16-3-2017 & 35/26-3-2019).

### 2.1. Participants

Adult men were recruited through advertisements in local newspapers. If interested, potential participants attended a short information session to receive additional study details and to determine eligibility. Healthy men between the ages of 25–50 years with a fasting blood glucose level < 125 mg/dL were eligible for participation [31]. Individuals were excluded from study participation if they were active tobacco smokers, diagnosed with a chronic disease, or taking medication to treat a chronic condition. During the initial meeting, a health history questionnaire was completed, and a fasting blood sample was collected for the assessment of blood glucose (Contour NEXT/ASCENSIA monitor). A total of 23 participants (27–47 years) were eligible for participation and completed the study requirements. All participants provided informed consent. As previously described, an a priori power calculation was not completed due to the lack of relevant published work related to the larger clinical trial [30].

### 2.2. Assessment of Dietary Intake

Eligible participants were asked to complete an assessment of dietary intake over 7 consecutive days. During the 7-day period, participants were asked to continue their normal dietary routine and to record all caloric intake for each 24-h period. A study investigator called each participant at 22:00 each evening during the 7-day period to retrieve daily food intake data via dietary recall. To get the most accurate information, the investigator asked additional questions about food preparation, ingredients, serving sizes, and dressings and/or other additives. Dietary intake data were analyzed by a trained investigator using the Nutritionist Pro (Version 8.1.0, Axxya Systems LLC., Redmond, WA, USA) software.

### 2.3. Anthropometry and Body Composition

On a day during the 7-day dietary intake measurement period, participants completed an assessment of anthropometry. To calculate body mass index, height and weight were measured using a stadiometer and scale, respectively. Body surface area was calculated using the Du Bois formula (BSA = 0.007184 ∗ Height^0.725^ ∗ Weight^0.425^) using height (cm) and weight (kg) as previously reported [32]. Waist and hip circumference were measured in triplicate by a trained investigator using a measuring tape. Body composition analysis was conducted using dual-energy X-ray absorptiometry (DXA) according to a procedure described previously [33]. Percent body fat and fat free mass of each participant were recorded for analysis.

### 2.4. Experimental Protocol

The experimental protocol procedures were completed at the Biomedical Research Foundation at the Academy of Athens. Participants arrived at the laboratory at 07:30 following an overnight fast and after refraining from exercise, alcohol, and passive smoking for the 72 h prior to the experimental testing. At 08:00, participants consumed a meal consisting of 50% carbohydrates, 20% protein, and 30% fat to normalize the thermic effect of food [34]. The total caloric value of the meal corresponded to 15% of each participant’s total daily energy requirement that was calculated using a method previously described [35]. At 09:00, participants were provided with a telemetric pill that recorded their core temperature in minute averages for the entire duration of the experimental protocol [1]. Thereafter, participants were asked to rest in a seated or supine position for approximately 60 min. Near the end of the resting period, blood pressure was measured using an Aneroid sphygmomanometer (Medisave, Weymouth, UK) [36].

#### 2.4.1. Cold Exposure Protocol

Immediately following the resting period, participants were asked to dress in specific clothing (long-sleeve sweatshirt and sweatpants) that provided a 0.49 clo level of insulation [1]. Thereafter, participants were asked to remain in a cold room (maintained at 16.7 ± 0.6 °C, 49 ± 11.1% relative humidity) while resting in a seated position for 120 min. An investigator entered the room every 15 min during the cold exposure period to conduct a shivering assessment [37]. Specifically, at each assessment the participant was categorized as: (1) no shivering (no tension of muscles reported), (2) mild shivering (slight muscle tonus of the masseter muscle), (3) moderate shivering (observable shivering of the proximal muscles), or (4) severe shivering (uncontrolled whole-body shivering) [37]. When participants experienced moderate or severe shivering, a blanket was provided for a 15-min period. Shivering intensity data were collected for 20 out of the 23 participants who completed the study protocol.

#### 2.4.2. PET/CT Examination

Brown adipose tissue activity was assessed immediately following cold exposure using positron emission tomography and computed tomography (PET/CT)—currently considered the gold standard method of BAT activity assessment in humans [38]. Each participant was injected with ^18^F fluorodeoxyglucose (^18^F-FDG) after the first hour of cold exposure. The dosage of injected ^18^F-FDG was 200–247 MBq depending on participant body weight. The PET/CT device obtained images from the base of the skull to the upper third of the thigh. Measurements of BAT were obtained from the left and right side of the supraclavicular area and along the spine. Seven bed positions at approximately 6 min per position were required for analysis. The maximum standardized uptake value (SUV_max_) in BAT was reported for each participant. As described previously, BAT was considered active, and participants were categorized as BAT-positive (BAT_positive_) if SUV_max_ values were greater than or equal to 1.5 g/mL [30]. Conversely, participants were categorized as BAT-negative (BAT_negative_) if SUV_max_ values were <1.5 g/mL. Scans obtained from PET/CT were examined independently by two experienced physicians [12]. Maximum standardized uptake value in BAT was normalized to lean mass (SUV_lean_) using the following equation (SUV_lean_ = SUV_max_ ∗ lean body mass/body mass) [31].

### 2.5. Statistical Analysis

Normality tests (Shapiro–Wilk) revealed that the majority of dietary intake variables followed a normal distribution. Participant characteristics were compared between BAT_negative_ and BAT_positive_ participants using independent-sample *t*-tests. Between-group comparisons of dietary intake variables were conducted using a multivariate general linear model (i.e., analysis of variance and analysis of covariance) both unadjusted and adjusted for age. Group (i.e., BAT_negative_ and BAT_positive_) was included as a fixed factor in both unadjusted and adjusted models, whereas age was included as a continuous covariate in the adjusted model. Among BAT_positive_ participants, associations between cold-induced BAT activation (i.e., SUV_lean_), participant characteristics, and dietary intake variables were assessed using Pearson’s product-moment correlation.

To test the association between shivering status and BAT activation during cold exposure, a chi-square test for association was conducted. Given the small value (<5) for the calculated expected counts, Fisher’s exact test results were reported and used for interpretation [39]. Anthropometry and dietary intake differences between the non-shivering and shivering groups were assessed using independent-sample *t*-tests. Data were analyzed using SPSS version 29 [40]. Statistical significance was set at *p* < 0.05 and all tests were two-sided.

## 3. Results

### 3.1. General Description of Study Participants

The study sample included 23 male participants with a mean age of 36.9 ± 5.2 years. Mean body weight, BMI, and waist circumference of the participants were 98.0 ± 25.7 kg, 29.6 ± 6.0 kg/m^2^, and 102.9 ± 16.7 cm, respectively. Body composition analysis included an assessment of participants’ percent body fat (29.6 ± 8.9%) and fat free mass (67.6 ± 11.5 kg). Participants had a mean systolic and diastolic blood pressure of 123.2 ± 9.9 mmHg and 82.8 ± 7.7 mmHg, respectively. Additional participant characteristics are presented in Table 1.

### 3.2. Cold-Induced Brown Adipose Tissue Activation

Twelve of 23 (52%) participants achieved an SUV_max_ greater than or equal to 1.5 g/mL following cold exposure and were categorized as BAT-positive (BAT_positive_). The BAT_positive_ participants were significantly younger (*p* = 0.046), showed a trend toward having a lower systolic blood pressure (*p* = 0.079), but were not significantly different in body surface area (BSA), BMI, waist circumference, percent body fat, fat free mass, diastolic blood pressure, or fasting blood glucose (*p* ≥ 0.177) when compared to participants who did not achieve sufficient levels of cold-induced BAT activation (SUV_max_ < 1.5 g/mL; BAT_negative_) (Table 1).

### 3.3. Comparison of Dietary Intake Variables Between BAT_negative_ and BAT_positive_ Participants

The BAT_negative_ participants consumed an average of 281.2 kcal/day more than BAT_positive_ participants, yet this difference was not deemed statistically significant (*p* = 0.242). The majority of additional kcal consumed by BAT_negative_ participants were in the form of carbohydrate (40.8 g = 163.2 kcal). Protein (13.8 g = 55.2 kcal) and fat (6.1 g = 54.9 kcal) were also consumed in greater quantities per day, on average, among BAT_negative_ compared to BAT_positive_ participants, although these differences were not statistically significant (*p* ≥ 0.271). Dietary intake data are summarized in Table 2. Differences in dietary intake between groups remained nonsignificant after adjustment for age, *F*(1, 20) ≤ 2.26, *p* ≥ 0.15, partial η^2^ ≤ 0.10.

### 3.4. Associations Between Cold-Induced BAT Activation (SUV_lean_) and Dietary Intake Among BAT_positive_ Participants

**Figure 1:**
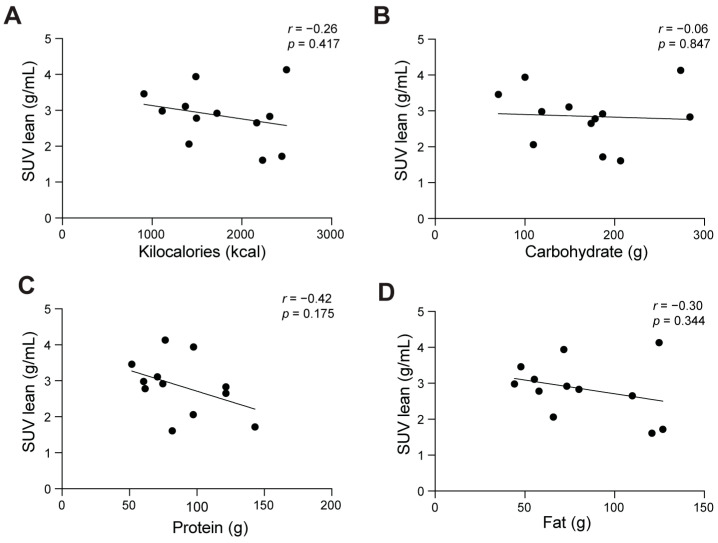
Relationship between level of cold-induced brown adipose tissue (BAT) activation normalized to lean mass and dietary intake variables among BAT_positive_ participants (*n* = 12). Panel (**A**) SUV_lean_ vs. Kilocalories; (**B**) SUV_lean_ vs. Carbohydrate; (**C**) SUV_lean_ vs. Protein; (**D**) SUV_lean_ vs. Fat. Participants who achieved BAT activation (maximum SUV of greater than or equal to 1.5 g/mL) were categorized as BAT_positive_. Associations were assessed using Pearson’s product-moment correlation. Maximum standardized uptake value normalized to lean mass (SUV_lean_).

BAT_positive_ participants had a mean SUV_lean_ of 2.8 ± 0.8 g/mL. SUV_lean_ was positively associated with height (*r*(10) = 0.613, *p* = 0.034), but was not significantly associated with any other anthropometric variable (*p* ≥ 0.179). Associations between SUV_lean_ and dietary intake variables were initially assessed using Pearson’s product-moment correlation. There were no statistically significant associations between SUV_lean_ and dietary intake among BAT_positive_ participants (*p* ≥ 0.175). In general, however, food intake, such as protein (*r*(10) = −0.419, *p* = 0.175) and saturated fat (*r*(10) = −0.406, *p* = 0.190) tended to decrease as SUV_lean_ values increased. Figure 1 includes scatterplots that describe associations between SUV_lean_ and kilocalories, carbohydrate, protein, and fat among BAT_positive_ participants. Pearson’s partial correlation analyses revealed that associations between cold-induced BAT activation and dietary intake variables remained nonsignificant after controlling for height (*rpartial*(9) ≤ −0.428, *p* ≥ 0.189).

### 3.5. Comparison of Cold-Induced Shivering Intensity Between BAT_negative_ and BAT_positive_ Participants

**Figure 2:**
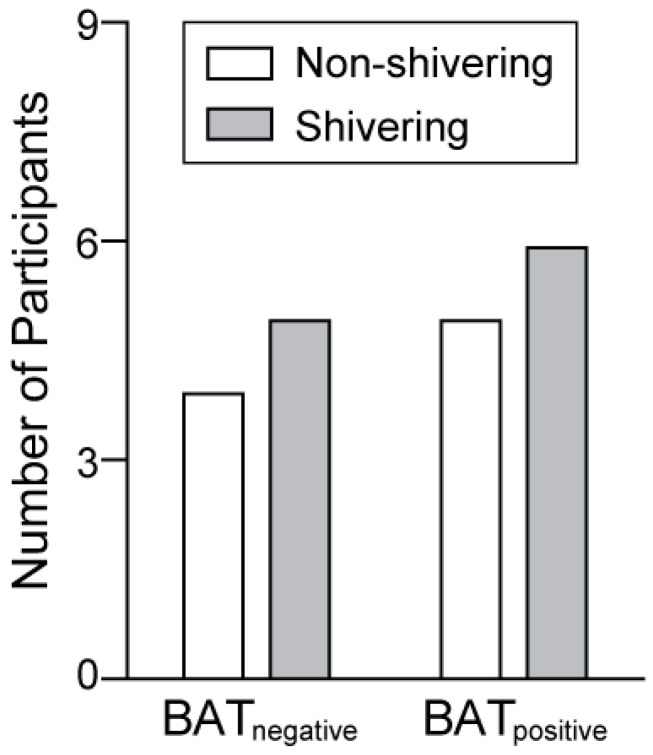
Shivering status during 120 min of cold exposure. Participants who achieved brown adipose tissue activation (maximum standardized uptake value of greater than or equal to 1.5 g/mL) were categorized as brown adipose tissue positive (BAT_positive_). Participants who did not achieve brown adipose tissue activation (maximum standardized uptake value less than 1.5 g/mL) were categorized as BAT_negative_. Participants who did not shiver during cold exposure were categorized as non-shivering, whereas participants who experienced mild, moderate, or severe shivering during cold exposure were categorized as shivering. Fisher’s exact test results revealed no association between shivering status and BAT activity (*p* = 0.964).

Fisher’s exact test was conducted to assess the association between shivering intensity and BAT activation (i.e., BAT_negative_ or BAT_positive_) during cold exposure. Participants who did not shiver during the 2-h cold exposure period were categorized as non-shivering, whereas participants who experienced any shivering (i.e., mild, medium, or high intensity) were categorized as shivering. Shivering status was assessed at each time point during cold exposure (i.e., 15, 30, 45, 60, 75, 90, and 120 min) and cumulatively during the entire cold exposure period—non-shivering (*n* = 9) vs. shivering (*n* = 11) at any point during cold exposure. There were no significant associations between shivering and BAT activation at 15, 30, 45, 60, 75, 90, and 120 min during cold exposure (*p* ≥ 0.670). Figure 2 shows the total number of non-shivering and shivering participants among the BAT_negative_ and BAT_positive_ groups (*p* = 0.964).

### 3.6. Comparison of Anthropometry and Dietary Intake Variables Between the Non-Shivering and Shivering Groups

An independent-samples *t*-test was conducted to assess the differences in anthropometry and dietary intake variables between the shivering and non-shivering groups. The shivering group tended to be shorter (*p* = 0.056), have a lower waist-to-hip ratio (*p* = 0.097), and a smaller BSA (*p* = 0.168) when compared to the non-shivering group, but these comparisons were not statistically significant. Kilocalories, carbohydrate, total fiber, total sugar, protein, fat, saturated fat, monounsaturated fat, polyunsaturated fat, omega-6 fatty acids, omega-3 fatty acids, and omega-6/omega-3 ratio were not significantly different between the non-shivering and shivering groups (*p* ≥ 0.173).

## 4. Discussion

The possibility of habitual dietary patterns influencing BAT activity presents an interesting opportunity for the development of dietary strategies that specifically target BAT activity for body weight regulation and to improve metabolic health. Currently, this topic of investigation has not been adequately explored, which prompted us to conduct the study presented herein. We compared dietary intake, collected over 7 consecutive days, among adult men with different levels of cold-induced BAT activation. We also assessed the relationship, among individuals categorized as BAT_positive_ (SUV_max_ ≥ 1.5 g/mL) between dietary factors and the extent to which BAT was activated in response to cold exposure. Finally, dietary intake and BAT activity were assessed in relation to shivering status during cold exposure. In this study, 52% (12/23) of participants had an SUV_max_ value of ≥1.5 g/mL and, therefore, were considered BAT_positive_. When the BAT_positive_ group was compared to the BAT_negative_ group, no differences in dietary intake variables were observed after both unadjusted and age-adjusted analyses. Similarly, among only the BAT_positive_ participants, BAT activity normalized to fat free mass (i.e., SUV_lean_) was not associated with any of the examined dietary intake variables. These nonsignificant associations remained after controlling for height. Dietary intake and BAT activity were similar among participants categorized as non-shivering or shivering during cold exposure. These findings support the contention that habitual dietary intake has a negligible influence on BAT activity among adult men.

Our results are consistent with Sanchez-Delgado et al. [23] and Maliszewska et al. [22] who reported no associations between BAT activity and habitual energy intake variables. In the previous studies, habitual energy intake was assessed over a 3-day period and BAT activity was measured using either PET/CT or PET/MR (i.e., magnetic resonance). Despite the lack of differences in habitual dietary intake, BAT_positive_ participants from our study cohort were significantly younger when compared to the BAT_negative_ group and, although not significant, tended to weigh less, have a lower BMI, a smaller waist circumference, and a higher systolic blood pressure. These findings are similar to Maliszewska et al. [22] who reported a significantly higher age, BMI, and visceral adipose tissue mass among BAT_negative_ versus BAT_positive_ participants. These results suggest that other factors, such as age, body weight, and percent body fat, may be more likely to influence BAT activity, as previously reported [41], rather than habitual dietary intake. It is, however, important to note that if the intention is to specifically stimulate BAT activity by manipulating habitual dietary intake, it may be useful to (1) include specific food or food items that have been shown to acutely stimulate BAT activity [18,19,42], and/or (2) modify habitual dietary intake to promote weight loss and general health. The latter may have an indirect influence on BAT activation given that individuals with a lower body weight and percent body fat have been reported to have higher BAT activation when compared to those with a higher body weight and percent body fat [41]. Nevertheless, this topic should be further investigated given that recent work has continued to emphasize the potential link between age-associated changes in BAT and longevity [43].

To add additional depth to the investigation of associations between habitual dietary intake and BAT activation, some have explored the quality of macronutrients in addition to quantity [22,27]. For example, both mono and polyunsaturated fatty acid intake have been linked to BAT activity in animal and human studies. Takahashi and Ide [27] reported that rats fed a high-fat diet rich in polyunsaturated fatty acids had an increased expression of UCP1 mRNA in BAT that was greater in rats fed a diet rich in omega-3 compared to omega-6 polyunsaturated fatty acids. In humans, it was recently reported that monounsaturated fatty acid intake and omega-6 polyunsaturated fatty acid intake were higher in BAT_negative_ participants when compared to their BAT_positive_ counterparts [22]. The authors suggested that dietary omega-6 fatty acid intake should be considered as a potential mechanism regulating BAT activity. Our results are not consistent with those reported by Maliszewska et al. [22]. We found no significant differences in the intake of monounsaturated fatty acids, polyunsaturated fatty acids, omega-3 fatty acids, omega-6 fatty acids, or omega-6/omega-3 ratio between the BAT_negative_ and BAT_positive_ groups. It is clear that discrepancies remain and that more work should be conducted to further elucidate the impact of reducing omega-6 fatty acid intake on BAT activity.

Eleven participants in our study experienced either mild or moderate shivering during the 120-min cold exposure period. Given that previous research has not shown a consistent relationship between shivering onset and BAT activity [44], we aimed to further investigate this relationship with consideration of habitual dietary intake. Prior to quantification of BAT, Sardjoe Mishre et al. [44] subjected individuals to 120 min of cold exposure and found that shivering threshold time was positively associated with BMI, lean mass, and fat mass in women only, whereas BSA was positively associated with shivering threshold in both sexes. Interestingly, it was also reported that shivering threshold time was not found to be associated with BAT volume or activity in both men and women [44]. In our study, we reported only whether or not shivering occurred during cold exposure and found that dietary intake variables were similar between the non-shivering and shivering participants. Moreover, our findings are consistent with Sardjoe Mishre et al., [44] given that we did not observe an association between shivering status and BAT activity. The participants who did shiver tended to be of a smaller stature. These findings suggest that habitual dietary intake and BAT activity among adult men are not directly related to shivering status during cold exposure. Similar to above, and as mentioned by others, it is more likely that variables such as body size and composition have a greater influence on shivering when compared to other variables such habitual dietary intake and BAT activation.

Limitations of our study include the method to assess dietary intake, based on self-reported dietary records that have been shown to underestimate total energy expenditure [45]. To strengthen the robustness of our dietary data, we extended the period of dietary intake assessment to 7 consecutive days, rather than the commonly used 3-day recording period. It is also important to note that, despite using a gold standard technique for the quantification of BAT activity, our sample size is small and is limited to adult male participants. Future work would benefit from studying a larger, more diverse sample and by incorporating additional control in relation to the assessment of dietary intake, which may include a dietary intervention. Finally, all participants were living in Greece at the time of data collection and were more likely to consume foods common in the Mediterranean, thus these findings may not be generalizable to other populations.

## 5. Conclusions

Habitual dietary intake was similar between adult men with and without cold-induced BAT activation. Further, among the participants with detectable BAT activity, there was no association between the extent to which BAT was activated (normalized to lean mass) and dietary intake variables assessed over a 7-day period. Our results support that BAT activity and shivering during cold exposure are more strongly related to variables such as age and body size or composition rather than habitual dietary intake. If the intention is to refine dietary intake for the purpose of stimulating BAT activity, we suggest incorporating specific food or food items that have been shown to acutely stimulate BAT activity, and/or modify habitual dietary intake to promote weight loss and general health. We conclude that habitual dietary intake likely has a negligible influence on BAT activity among adult men.

## Figures and Tables

**Table 1:** Participant Characteristics.

Variable	Total (*n* = 23)	BAT_negative_ (*n* = 11)	BAT_positive_ (*n* = 12)	*p*-Value ^a^
Age (years)	36.9 ± 5.2	39.1 ± 4.1	34.8 ± 5.4	0.046 ^b^
Height (cm)	181.1 ± 6.8	182.2 ± 7.5	180.0 ±6.2	0.434
Weight (kg)	98.0 ± 25.7	105.0 ± 30.6	91.6 ± 19.4	0.234
Body surface area (m^2^)	2.2 ± 0.3	2.2 ± 0.3	2.1 ± 0.2	0.258
BMI (kg/m^2^)	29.6 ± 6.0	31.1 ± 6.7	28.2 ± 5.3	0.257
Healthy weight, *n* (%)	6 (26.1)	2 (18.2)	4 (33.3)	0.640 ^c^
Overweight/obese, *n* (%)	17 (73.9)	9 (81.8)	8 (66.7)	
Waist circumference (cm)	102.9 ± 16.7	106.7 ± 19.2	99.5 ± 14.0	0.309
Waist to hip ratio	0.9 ± 0.1	0.9 ± 0.1	0.9 ± 0.1	0.297
Percent body fat (%)	29.6 ± 8.9	30.4 ± 8.3	28.8 ± 9.6	0.660
Fat free mass (kg)	67.7 ± 11.5	71.1 ± 15.0	64.6 ± 6.0	0.177
Systolic blood pressure (mmHg)	123.2 ± 9.9	127.0 ± 7.3	119.8 ± 11.0	0.079
Diastolic blood pressure (mmHg)	82.8 ± 7.7	84.9 ± 8.2	80.9 ± 6.9	0.220
Fasting blood glucose (mg/dL) ^d^	89.5 ± 7.8	90.7 ± 7.1	88.4 ± 8.5	0.504

Participants who achieved sufficient brown adipose tissue activation (maximum standardized uptake value of greater than or equal to 1.5 g/mL) immediately following cold exposure were categorized as brown adipose tissue positive (BAT_positive_). Participants who did not achieve a sufficient level of cold-induced brown adipose tissue activation (maximum standardized uptake value less than 1.5 g/mL) were categorized as BAT_negative_. Data are presented as mean ± standard deviation. Body mass index (BMI). ^a^ With the exception of BMI category, data were compared between BAT_negative_ and BAT_positive_ participants using an independent-samples *t*-test. ^b^ Indicates a statistically significant difference between BAT_negative_ and BAT_positive_ participants. ^c^ Association between BMI category and BAT activation was assessed by conducting a chi-square test for association. ^d^ Fasting blood glucose data were collected from 21 participants.

**Table 2:** Dietary Intake Data.

Variable	Total (*n* = 23)	BAT_negative_ (*n* = 11)	BAT_positive_ (*n* = 12)	*p*-Value ^a^
Kilocalories	1897.7 ± 565.8	2044.4 ± 576.3	1763.2 ± 545.0	0.242
Carbohydrate (g)	189.2 ± 75.3	210.5 ± 82.5	169.7 ± 65.5	0.202
Total fiber (g)	18.0 ± 8.8	19.7 ± 9.3	16.4 ± 8.4	0.375
Total sugar (g)	70.1 ± 41.6	71.4 ± 44.5	68.9 ± 40.7	0.888
Protein (g)	94.8 ± 29.4	102.0 ± 30.2	88.2 ± 28.4	0.271
Fat (g)	84.5 ± 32.0	87.7 ± 34.5	81.6 ± 30.8	0.657
Saturated fat (g)	30.8 ± 14.7	32.6 ± 15.6	29.3 ± 14.3	0.600
Monounsaturated fat (g)	31.0 ± 12.6	32.4 ± 13.5	29.6 ± 12.1	0.603
Polyunsaturated fat (g)	12.9 ± 3.6	13.2 ± 4.1	12.5 ± 3.2	0.634
ω-6 (g)	11.1 ± 3.3	11.5 ± 3.7	10.8 ± 3.1	0.616
ω-3 (g)	1.2 ± 0.4	1.2 ± 0.4	1.2 ± 0.4	0.881
ω-6:ω-3 ratio	9.5 ± 3.2	9.3 ± 1.2	9.8 ± 4.4	0.755

Dietary intake values were calculated as the daily average over a 7-day period. Participants who achieved sufficient brown adipose tissue activation (maximum standardized uptake value of greater than or equal to 1.5 g/mL) immediately following cold exposure were categorized as brown adipose tissue positive (BAT_positive_). Participants who did not achieve sufficient levels of cold-induced brown adipose tissue activation (maximum standardized uptake value less than 1.5 g/mL) were categorized as BAT_negative_. Data are presented as mean ± standard deviation. ^a^ Data were compared between BAT_negative_ and BAT_positive_ participants using an independent-samples *t*-test.

## Data Availability

Inquiries or requests regarding participant data can be directed to the corresponding author.
